# Dark period transcriptomic and metabolic profiling of two diverse *Eutrema salsugineum* accessions

**DOI:** 10.1002/pld3.32

**Published:** 2018-02-22

**Authors:** Jie Yin, Michael J. Gosney, Brian P. Dilkes, Michael V. Mickelbart

**Affiliations:** ^1^ Department of Horticulture and Landscape Architecture Purdue University West Lafayette IN USA; ^2^ Department of Botany and Plant Pathology Purdue University West Lafayette IN USA; ^3^ Department of Biochemistry Purdue University West Lafayette IN USA

**Keywords:** differentially expressed genes, extremophile, fatty acid metabolism, metabolomics, single nucleotide polymorphisms, transcriptome, transgressive variation

## Abstract

*Eutrema salsugineum* is a model species for the study of plant adaptation to abiotic stresses. Two accessions of *E. salsugineum*, Shandong (SH) and Yukon (YK), exhibit contrasting morphology and biotic and abiotic stress tolerance. Transcriptome profiling and metabolic profiling from tissue samples collected during the dark period were used to investigate the molecular and metabolic bases of these contrasting phenotypes. RNA sequencing identified 17,888 expressed genes, of which 157 were not in the published reference genome, and 65 of which were detected for the first time. Differential expression was detected for only 31 genes. The RNA sequencing data contained 14,808 single nucleotide polymorphisms (SNPs) in transcripts, 3,925 of which are newly identified. Among the differentially expressed genes, there were no obvious candidates for the physiological or morphological differences between SH and YK. Metabolic profiling indicated that YK accumulates free fatty acids and long‐chain fatty acid derivatives as compared to SH, whereas sugars are more abundant in SH. Metabolite levels suggest that carbohydrate and respiratory metabolism, including starch degradation, is more active during the first half of the dark period in SH. These metabolic differences may explain the greater biomass accumulation in YK over SH. The accumulation of 56% of the identified metabolites was lower in F_1_ hybrids than the mid‐parent averages and the accumulation of 17% of the metabolites in F_1_ plants transgressed the level in both parents. Concentrations of several metabolites in F_1_ hybrids agree with previous studies and suggest a role for primary metabolism in heterosis. The improved annotation of the *E. salsugineum* genome and newly identified high‐quality SNPs will permit accelerated studies using the standing variation in this species to elucidate the mechanisms of its diverse adaptations to the environment.

**Abbreviations**
ABA: Abscisic acid
DEGs: differentially expressed genes
ESTs: expressed sequence tags
ET: ethylene
FDR: false discovery rate
GC/MS: gas chromatography‐mass spectrometry
JA: jasmonic acid
MeJA: methyl jasmonate
PAV: presence–absence variation
PDF: plant defensin gene
PMSR: peptide methionine sulfoxide reductase
QTL: quantitative trait loci
SH: *E. salsugineum* Shandong accession
SNP: single nucleotide polymorphism
VLCFA: very‐long‐chain fatty acids
YK: *E. salsugineum* Yukon accession

## 1 INTRODUCTION

*Eutrema salsugineum* (formerly *Thellungiella halophila*) is a model species for the study of plant stress tolerance (Amtmann, 2009; Griffith et al., 2007; Pilarska et al., 2016; Wong et al., 2005). The two most commonly studied accessions, Shandong (SH) and Yukon (YK), are native to the Yellow River region of China (Bressan et al., 2001; Inan et al., 2004) and the Yukon territories of Canada (Wong et al., 2005), respectively. These accessions contrast in cold tolerance (Lee, Babakov, de Boer, Zuther, & Hincha, 2012), water stress tolerance (MacLeod et al., 2014; Xu et al., 2014), and disease resistance (Yeo et al., 2014). In response to water stress, for example, YK accumulates more cuticular wax (Xu et al., 2014), exhibits delayed wilting due to higher leaf water content, and maintains a higher leaf area, as compared to SH (MacLeod et al., 2014). Some differences in adaptive mechanisms are linked to metabolism, such as a more pronounced increase in fructose and proline content after cold acclimation in YK compared to SH (Lee et al., 2012).

RNA‐seq has been used to identify differentially expressed genes (DEGs) that contribute to genotypic variation in response to physiological conditions (Bazakos et al., 2012; Huang et al., 2012; Stein & Waters, 2012; Wang, Gerstein, & Snyder, 2009). When comparing expression between polymorphic accessions of a species, RNA‐seq allows for the simultaneous identification and quantification of single nucleotide polymorphisms (SNPs; Nielsen, Paul, Albrechtsen, & Song, 2011; Wang et al., 2009). These data can later be utilized as genetic markers for linkage mapping (Lisec et al., 2008; Trick et al., 2012) and to assess allele‐specific expression (Oshlack, Robinson, & Young, 2010; Pickrell et al., 2010).

Plant metabolic profiling permits the simultaneous measurement of multiple intermediates and the products of biochemical pathways. Similar to RNA‐seq, metabolic profiling can be used to investigate the metabolic and physiological status of biological systems (Fiehn et al., 2000) and may provide biochemical bases for differences in growth and physiology (Meyer et al., 2007). Metabolite profiling has revealed correlations between particular metabolites and growth in *Arabidopsis thaliana* (Meyer et al., 2007), but it is not clear whether these metabolites are more universally linked to heterosis.

Novel genes and enzymes in plant metabolite biosynthetic pathways have been identified through combined transcriptome and metabolome analyses (Boke et al., 2015; Sumner, Lei, Nikolau, & Saito, 2015). In addition, this approach has provided insights into pathways that are affected by gene mutation (Masclaux‐Daubresse et al., 2014; Page et al., 2016; Satou et al., 2014), related to biotic (Gurkok, Turktas, Parmaksiz, & Unver, 2015; Liu et al., 2016), and abiotic stress responses (Bielecka et al., 2014; Hamanishi, Barchet, Dauwe, Mansfield, & Campbell, 2015).

Together, transcriptome profiling and metabolite profiling provide complementary experimental evidence to guide the construction of rational hypotheses for the biochemical basis of variation in growth. The goal of this study is to identify the metabolic and transcription bases for the growth differences between the two *E. salsugineum* accessions. We utilized contrasting genotypes to identify genetic differences, expression divergence, and metabolic compounds associated with observed phenotypic variation. The YK accession has a higher water‐use efficiency than SH (J Yin et al., manuscript in preparation). Among several traits that differ in these accessions is a distinct change in transpiration during the dark period. We therefore employed transcriptome and metabolic profiling to provide insight on the observed differences in these accessions and chose the dark period for tissue collection because of this observation. We obtained gene expression and metabolite concentration data from SH and YK accessions during the dark period by RNA‐seq and gas chromatography‐mass spectrometry (GC/MS), respectively. We utilized RT‐PCR to confirm DEGs implicated by our RNA‐seq experiment, validating 23 of 25 candidates. By mining the RNA‐seq experiment, we validated previously identified SNPs and discovered additional SNPs that differentiate these *E. salsugineum* accessions. We propose that observed differences in metabolite accumulation could contribute to differences in biomass.

## 2 MATERIALS AND METHODS

### 2.1 Plant material and growth conditions

Seeds of *E. salsugineum* SH and YK accessions were obtained from individual selfed plants. To generate SH × YK F_1_ seed, closed flower buds of YK plants were manually opened, anthers removed, and pollen from SH plants applied to the stigma. Multiple crosses (ca. 20) were made on a single plant, which was kept in isolation until seed set. Seeds from this plant were used for F_1_ experiments.

Seeds were stratified in the dark for 10 days at 4°C and then sown in a 4:1 mix of Fafard 52 Mix (transcriptome and first metabolome experiments) or Fafard 2 Mix (second metabolome experiment) soilless media (Conrad Fafard Inc., Agawam, MA, USA) and Turface calcined clay (Profile Products LLC, Buffalo Grove, IL, USA). Plants were grown in 50‐ml conical tubes (USA Scientific Inc., Ocala, FL, USA). A 0.5‐cm hole was drilled at the bottom of each tube where seeds were sown. Tubes were then closed with a mesh cap and placed cap‐side‐down to allow for subirrigation. Tubes were placed in a mist room for 12 days, at which point seedlings had reached the four‐leaf stage. Plants were then grown in a growth chamber (E15; Conviron, Pembina, ND, USA) set at 60% relative humidity under a 12‐hr photoperiod (approximately 230 μmol m^−2^ s^−1^ provided by both fluorescent and incandescent bulbs) with light and dark temperatures of 22 and 20°C, respectively. Irrigation water was a 3:1 mix of two water‐soluble fertilizers (15N–2.2P–12.5K and 21N–2.2P–16.6K; The Scotts Co., Marysville, OH, USA) to supply (in mg/L): 200 N, 26 P, 163 K, 50 Ca, 20 Mg, 1.0 Fe, 0.5 Mn and Zn, 0.24 Cu and B, and 0.1 Mo; 76% of the nitrogen was provided as nitrate. 93% sulfuric acid (Brenntag North America Inc., Reading, PA, USA) at 0.08 mg/L was mixed in irrigation water to maintain pH between 5.8 and 6.2. All plants were grown in the Purdue University Horticulture Plant Growth Facility (https://ag.purdue.edu/hla/Hort/Greenhouse/Pages/Default.aspx).

### 2.2 Transcriptome sequencing and analysis

For RNA extraction, tissue was collected in the middle of the dark period. Four biological replicates, each consisting of five whole rosettes of 4‐week‐old plants, were collected and immediately frozen in liquid nitrogen and stored at −80°C. Tissue was finely ground in liquid nitrogen using a mortar and pestle. Approximately 800 mg ground tissue was combined with 1 ml of TRIzol reagent (Gibco/BRL Life Technologies; Invitrogen, Carlsbad, CA, USA) and RNA was extracted according to the manufacturer's instructions. Genomic DNA was removed using the TURBO DNA‐free™ Kit (Ambion, Austin, TX, USA). Quality of RNA was estimated by the ratio of absorbance at 260 to 280 nm and 260 to 230 nm by spectrophotometer (DU 730; Beckman Coulter Inc., Indianapolis, IN, USA) with both ratios between 1.8 and 2.2. RNA was reverse‐transcribed into cDNA using the Poly(A) Purist protocol (Ambion).

The cDNA samples were fragmented into 300‐ to 500‐bp molecules, and sequencing libraries were constructed for the 454 GS‐FLX instrument (454 Life Sciences; Roche Company, Branford, CT, USA) at the Purdue University Genomics Facility (West Lafayette, IN, USA). One sequencing run was performed (Margulies et al., 2005). Raw sequences were trimmed to remove adaptor sequences, and an initial quality trimming was performed using GS De Novo Newbler (v2.5.3; default parameters). Trimmed reads were further trimmed using the FASTX‐Toolkit (Gordon & Hannon, 2010), with a minimum quality value of 12 and minimum read length of 50 bp. Trimmed reads were aligned to the Joint Genome Institute (JGI) *E. salsugineum* genome (Phytozome v9.1: *Thellungiella halophila*: http://www.phytozome.net/thellungiella.php; Yang et al., 2013) using the splice‐aware aligner GMAP v2012‐11‐27 with default parameters (Wu & Watanabe, 2005). Only uniquely mapped reads were used for transcript abundance estimates and single nucleotide polymorphism (SNP) calling.

SNPs were detected using mpileup from SAMtools v0.1.18 with mapping quality ≥15, and depth ≥3 (Li et al., 2009); 454 sequencing has a high error rate for detecting indels (Margulies et al., 2005), so only SNPs resulting from substitutions were retained. The two accessions are substantially inbred lines and should be homozygous at each base position. Hence, only monomorphic base positions within each accession were considered for detection of differences between the two accessions. Custom Perl scripts were used to remove SNPs (i) that were heterozygous within either accession, (ii) that were not biallelic between accessions, (iii) that were supported by fewer than three sequence reads, (iv) for which the alternative allele accounts for fewer than 10% of aligned reads, and (v) that were heterozygous between the SH accession and the JGI SH reference. If more than four SNPs were detected within a 100‐bp region using the VariantFiltration module from GATK v2.4.9 (McKenna et al., 2010), they were not included in the final SNP data set. SNPs that had a mpileup quality score 999 based on SAMtools were deemed “high‐quality” SNPs. Sanger sequencing data of the YK accession, available from the National Center for Biotechnology Information (NCBI) (Wong et al., 2005), were also aligned to the reference genome using SSAHA2 (Ning, Cox, & Mullikin, 2001). SNPs were called using SAMtools and filtered for clustered SNPs (four SNPs within 100‐bp region) using GATK as indicated. SNPs that were not biallelic or were heterozygous within YK were removed.

Genes were identified via a reference annotation‐based transcript assembly method using the Cufflinks package (Roberts, Pimentel, Trapnell, & Pachter, 2011; Trapnell et al., 2010). Reads from SH and YK were assembled separately and then merged using the *cuffmerge* command (Roberts et al., 2011; Trapnell et al., 2010). The *intersect* function within BEDTools v2.17.0 was used to identify genes not annotated in the JGI *E. salsugineum* genome (newly annotated genes). Same strandedness was not enforced when identifying newly annotated genes because of the nonstrand‐specific protocol for 454 library preparation. Newly annotated genes that are unique from or overlap genes annotated by Champigny et al. (2013) but are present in the JGI reference genome were also identified using the same method (Table S1).

The number of reads uniquely aligned to each gene was determined with htseq‐count within HTSeq v0.5.4p5 (http://www-huber.embl.de/users/anders/HTSeq/doc/count.html) using *union* mode. The bioconductor package “DESeq” v.1.14.0 was used to identify genes likely to be differentially expressed between SH and YK without biological replicates (Anders & Huber, 2010). Gene expression was normalized, and the significance threshold for differential expression was based on a 0.2 false discovery rate (FDR; Benjamini & Hochberg, 1995).

Genes were annotated by the best BLAST (Altschul, Gish, Miller, Myers, & Lipman, 1990) hit of *A. thaliana*. For *E. salsugineum* genes predicted by the JGI genome, annotation was taken from Phytozome v9.1 (Phytozome v9.1: *Thellungiella halophila*: http://www.phytozome.net/thellungiella.php, Yang et al., 2013). For newly identified genes, nucleotide databases of *A. thaliana* version TAIR 10 (Lamesch et al., 2012; TAIR10: ftp://ftp.arabidopsis.org/home/tair/Sequences/blast_datasets/TAIR10_blastsets/)*, A. lyrata* (Hu et al., 2011; Phytozome v9.1: *Arabidopsis lyrata*: http://www.phytozome.net/alyrata.php), and *S. parvula* (Dassanayake et al., 2011; *Thellungiella parvula* genome: http://www.thellungiella.org/data) were built and used for similarity searching by BLASTN v2.2.28 +  (Altschul et al., 1990). Genes were annotated by the best BLAST hit with the following threshold parameters: *E* ≤ 1^−30^; sequence identity ≥30%; sequence aligned ≥30% of query sequence.

### 2.3 Quantitative real‐time PCR

For quantitative real‐time reverse transcription–polymerase chain reaction (qRT‐PCR), tissue was collected as described for 454 sequencing, RNA was extracted using the RNeasy Plant Mini Kit (QIAGEN, Valencia, CA, USA), and genomic DNA was removed using the TURBO DNA‐free™ Kit (Ambion). The quality and quantity of mRNA were assessed using a NanoDrop 2000 (Thermo Fisher Scientific Inc., Wilmington, DE, USA). All samples were diluted to 200 ng/μl, and 260/280 and 260/230 ratios were between 1.8 and 2.2. cDNA was synthesized using a High‐Capacity cDNA Reverse Transcription Kit (Invitrogen). Primers were designed using Primer Express software (v3.0.1). Primer specificity was then estimated by BLASTN using the *E. salsugineum* genome with all primer pairs. Primer efficiency was tested for all pairs of primers. cDNA was diluted five times by a fivefold gradient and then used as template for qRT‐PCR, and the threshold cycles (*C*_*T*_) were regressed against cDNA concentration (log). Slope of the regression line was estimated, and the efficiency was calculated as 10^−(1/slope)^−1. For genes expressed in both accessions, primer efficiency was between 80 and 110% in both accessions. For genes that were only expressed in one accession based on RNA‐seq data, primer efficiency was tested on both accessions, but only the accession with detected expression exhibited efficiency between 80 and 110%. Table S2 contains all primer sequences except gene XLOC_004723, for which acceptable qRT‐PCR primers could not be designed.

All qRT‐PCR reactions were conducted in StepOnePlus™ Real‐Time PCR Systems (Applied Biosystems, Invitrogen). Relative gene expression of target genes was quantified by the Δ*C*_*T*_ method (Livak & Schmittgen, 2001). Relative gene expression was calculated as:

$$\begin{array}{cc} & \text{Relative expression}\;={\left(1+E\right)}^{-\Delta C_{T}}\\ & \;\;\;\Delta C_{T}=C_{T,X}-C_{T,R} \end{array}$$

where *E* is the primer efficiency for each pair of qRT‐PCR primers. *C*_*T,X*_ and *C*_*T,R*_ is the threshold cycle of the target gene and the reference gene *Actin2* (Thhalv10020906 m.g), respectively.

### 2.4 Metabolite profiling and data analysis

Two metabolite profiling experiments were conducted. One experiment was performed using the same rosette tissue used for RNA‐seq analysis (see above). A second metabolite profiling experiment was performed using tissue from SH, YK, and YK × SH F_1_ plants with three replicates of five pooled plants per replicate. In both cases, identical extraction, derivatization, and analysis methods were used. Approximately 800 mg of frozen ground tissue was incubated in methanol at 65° in 1.75‐ml tubes and centrifuged at 13,300 r/min. The supernatant, containing polar molecules, was decanted into a new tube. Chloroform was added to the pellet and incubated at 37°C for 15 min to solubilize nonpolar metabolites. Samples were then dried at room temperature for about 6 hr (polar) and 2 hr (nonpolar) in a centrifuge at 1,725 r/min and 30 μM Hg vacuum. Samples were stored at −80°C until being sent to the Metabolomics Center at the University of Illinois (http://www.biotech.uiuc.edu/metabolomics/). Samples were measured by gas chromatography‐mass spectrometry (GC‐MS; Agilent 6890 N/5973 MSD, Palo Alto, CA, USA) after purification and trimethylsilylation with MSTFA (*N*‐methyl‐*N*‐trimethylsilyl‐trifluoroacetamide; Gullberg, Jonsson, Nordström, Sjöström, & Moritz, 2004; Singh, Ulanov, Li, Jayaswal, & Wilkinson, 2011). Data were analyzed by peak identification via comparison with spectra from standards, and relative concentrations of metabolites were obtained by comparison with internal standard peak area (Singh et al., 2011).

Pairwise comparisons within each experiment were performed by two‐tailed *t* tests between SH and YK (experiments 1 and 2), and SH or YK and YK × SH F_1_ (experiment 2). In the second experiment, multiple comparisons among all three genotypes were conducted by Tukey's studentized range test (Tukey, 1949). A *t* test of F_1_ against the mid‐parental average of SH and YK was done using the variance estimated from F_1_ hybrids. Within each experiment, genotype was treated as the only main factor. A nested analysis of variance (ANOVA) was also conducted using data from the two experiments. In the nested analysis, experiment and genotype were the two main factors. The experiment by genotype interaction was not included in the model. All identified metabolites are presented in Table S3.

### 2.5 Pathway analysis

Pathway analysis was conducted on the annotated DEGs identified in transcriptome profiling and metabolites that differed in the same direction between accessions in both metabolite experiments. The fold difference between SH and YK was used to indicate up‐ or downregulation in YK compared to SH. The list of DEGs or metabolites with the fold changes were imported to MapMan software (Thimm et al., 2004). Significant pathways in which genes or metabolites were divergent from a 50/50 up/downregulation were identified using an uncorrected Wilcoxon signed‐rank test (Wilcoxon, 1945).

## 3 RESULTS

### 3.1 Novel genes and single nucleotide polymorphisms (SNPs) were identified by transcriptome sequencing

Whole rosettes of 4‐week‐old YK and SH plants grown in a 12‐hr:12‐hr light–dark cycle were harvested in the middle of the dark period. Libraries of cDNA isolated from these rosettes were sequenced, and reads were aligned to the *E. salsugineum* SH reference genome (Yang et al., 2013). More than 1 million cDNA sequence reads, 95% of which aligned to the reference genome, were used for a reference‐directed assembly of the transcriptome (Table S4), identifying 17,888 expressed genes (Tables S1 and S5). Of these, 65 genes were novel and not predicted in the reference genome (Yang et al., 2013) nor detected in a previous transcriptome analysis (Champigny et al., 2013). Only 20 of these 65 genes have annotated orthologs in the related species *A. thaliana*,* A. lyrata*, and/or *Schrenkiella parvula* (Table S1). Presence–absence variation (PAV), defined as zero reads aligned to one of the two parents, was observed for 18.5% of the detected expressed genes with roughly equal numbers of genes detected only in SH or YK (Table S5).

The transcript assemblies were processed to detect SNPs between SH and YK; 42% of shared genes contained a total of 14,808 SNPs, of which 4,873 were deemed “high quality” (Table 1; Table S6). Of the low and high stringency SNPs detected in our experiment, 73% (10,883 positions) and 79% (3,861 positions), respectively, were also identified by Champigny et al. (2013). We also compared our SNPs to available Sanger sequencing of cDNA clones from the YK accession (Wong et al., 2005) and identified 468 putative SNPs with reference to the SH reference genome. Of these, 441 have corresponding sequence data in the YK transcriptome we assembled and 88% (388 SNPs) had the same sequence variation in our assembly and the YK Sanger sequencing data (Table S6).

**Table 1:** Summary of single nucleotide polymorphisms (SNPs) that differentiate *Eutrema salsugineum* Shandong and Yukon accessions

Number of genes with SNPs	6,182
Number of SNPs in genes	14,808
Number of high‐quality SNPs	4,873
Number of SNPs identified by Champigny et al. (2013)	47,317
Number of SNPs present in Champigny et al. (2013)	10,883
Number of SNPs unique from Champigny et al. (2013)	3,925

Quality SNP satisfies technical and biologically relevant filtering criteria as discussed in the Materials and Methods.

### 3.2 Transcriptome profiling and qRT‐PCR identify differentially expressed genes (DEGs) between SH and YK

To assess gene expression differences between SH and YK, we determined the number of reads aligned to each gene. Less than 0.2% (thirty‐one genes) of the expressed genes were identified as candidate DEGs between SH and YK (Table 2). Sixteen of these thirty‐one candidates do not have homologous genes in *A. thaliana*,* A. lyrata*, or *S. parvula*, and twenty have been annotated in the reference genome (Table 2). Of those with homologs in one of these species, none have been associated with previously reported trait differences in SH and YK. Gene ontology (GO) enrichment analysis was not appropriate, given the small number of DEGs.

**Table 2:** Differently expressed genes (DEGs) of *Eutrema salsugineum* Shandong (SH) and Yukon (YK) accessions

*Eutrema* gene locus	SH	YK	Fold difference (log_2_YK/SH)	Fold difference in qRT	*A. thaliana* best blast hit	Gene description
Thhalv10021382 m.g	4	87	4.4	12.5	AT1G76810	Eukaryotic translation initiation factor 2 (eif‐2) family protein
Thhalv10000662 m.g	46	429	3.2	4.4	AT5G44420	Plant defensin 1.2A
Thhalv10015083 m.g	34265	2494	−3.8	−5661.5		
Thhalv10011087 m.g	1445	327	−2.1	n.s.	ATCG00480	ATP synthase subunit beta
XLOC_024729	237	25	−3.2	−2.0		
Thhalv10015718 m.g	0	48	Absent in SH	Not detected in SH	AT5G56920	Cystatin/monellin superfamily protein
Thhalv10029390 m.g	0	83	Absent in SH	Not detected in SH	AT4G11000	Ankyrin repeat family protein
Thhalv10021522 m.g	1	244	7.9	144.9	AT3G16660	Pollen Ole e 1 allergen and extensin family protein
Thhalv10014718 m.g	1	86	6.4	11.8	AT5G07470	Peptide methionine sulfoxide reductase 3
Thhalv10002969 m.g	61	0	Absent in YK	−58.3	AT3G62210	Putative endonuclease or glycosyl hydrolase
Thhalv10009345 m.g	58	0	Absent in YK	Not detected in YK		
Thhalv10014264 m.g	53	0	Absent in YK	Not detected in YK	AT4G01560	Ribosomal RNA processing Brix domain protein
Thhalv10000285 m.g	179	0	Absent in YK	Not detected in YK	AT4G20095	Protein of unknown function (DUF626)
Thhalv10022943 m.g	148	0	Absent in YK	Not detected in YK		
Thhalv10023491 m.g	47	0	Absent in YK	Not detected in YK	AT3G61100	Putative endonuclease or glycosyl hydrolase
Thhalv10018393 m.g	70	1	−6.1	1.7	AT1G70160	Unknown protein
XLOC_005768	0	99	Absent in SH	Not detected in SH		
XLOC_017575	50	0	Absent in YK	Not detected in YK		
XLOC_003055	55	0	Absent in YK	Not detected in YK		
XLOC_024727	53	0	Absent in YK	Not detected in YK		
XLOC_003052	318	1	−8.3	−257.7		
XLOC_008740	135	3	−5.5	Not detected in YK		
XLOC_020731	76	0	Absent in YK	Not detected in YK		
XLOC_017573	246	0	Absent in YK	Not detected in YK		
XLOC_015175	75	0	Absent in YK	Not detected in YK		
Genes with no specific primers available						
Thhalv10022994 m.g	0	46	Absent in SH	Not tested	AT2G15220	Plant basic secretory protein (BSP) family protein
Thhalv10022932 m.g	74	1	−6.2	Not tested		
Thhalv10014933 m.g	295	32	−3.2	Not tested	AT5G59870	Histone H2A 6
Thhalv10019398 m.g	6	217	5.2	Not tested	AT5G44430	Plant defensin 1.2C
Thhalv10029246 m.g	0	47	Absent in SH	Not tested	AT4G11000	Ankyrin repeat family protein
XLOC_004723	187	16	−3.5	Not tested	ATCG01020	Ribosomal protein L32

To confirm DEGs based on RNA‐seq, we designed qRT‐PCR primers that matched unique positions in the reference genome based on BLAST analysis (Altschul et al., 1990). Unique primers could not be designed for five genes (Thhalv10022994 m.g, Thhalv10022932 m.g, Thhalv10014933 m.g, Thhalv10019398 m.g, and Thhalv10029246 m.g) due to paralogs with high sequence similarity, and no acceptable primer pair was identified for XLOC_004723. In total, qRT‐PCR data confirmed our RNA‐seq data for 23 of 25 genes. For the five DEGs that had no close paralogs and at least four reads in both SH and YK, expression differences based on RNA‐seq were confirmed by qRT‐PCR in four of five genes (Table 2). Among the 20 genes that had fewer than four reads in either SH or YK, expression differences by qRT‐PCR was consistent with RNA‐seq data in 19 (Table 2). In addition, when the low accession had fewer than four reads, there was no amplification in 16 of 20 cases (Table 2).

### 3.3 Metabolite profiling reveals higher accumulation of fatty acids and amino acids in YK and enhanced soluble carbohydrate accumulation in SH

To identify potential metabolites and metabolic pathways that contribute to phenotypic and physiological differences between SH and YK, metabolite profiling was conducted in two independent experiments. In one experiment, metabolite concentrations in F_1_ plants of a YK × SH cross were also determined.

Concentrations of free fatty acids and long‐chain fatty acid derivatives were higher in YK than SH (Table 3; Table S3). The concentration of ferulic acid was also greater in YK, indicating a potential for greater suberin and/or cutin accumulation in the YK accession.

**Table 3:** Metabolite profiling differences between *Eutrema salsugineum* Shandong (SH) and Yukon (YK) accessions. Metabolites for which tissue concentrations were different between *E. salsugineum* Shandong (SH) and Yukon (YK) accessions in one or two experiments and/or different based on a nested analysis of the two experiments

Metabolite	Experiment 1		Experiment 2		*p‐*Value		
	SH	YK	SH	YK	E1	E2	N
Carbohydrates							
2‐keto‐gluconic acid	1012.0	526.4	352.9	470.8	n.s.	*	n.s.
2PGA	2.1	0.0			**		
α‐ketoglutaric acid	83.2	41.3	66.5	61.2	*	n.s.	*
Arabitol	45.4	60.9	12.9	19.5	n.s.	*	n.s.
Ascorbic acid	19.6	11.8	25.0	10.4	n.s.	**	**
Citric acid	6722.6	17762.7	14347.0	30526.0	n.s.	**	**
Digalactosylglycerol	2007.8	618.8	344.5	82.9	n.s.	***	n.s.
Disaccharide	9941.3	8651.1	10713.7	3862.7	n.s.	***	**
Erythritol	27.6	39.1	22.1	34.3	n.s.	*	*
Erythrose			976.8	591.3		*	
Fructose	65604.0	9500.3	14987.4	1284.4	***	***	***
Fumaric acid	232.3	429.0	111.5	263.1	n.s.	***	**
Galactaric acid			3.4	8.5		*	
Galactose	7730.3	4432.0	32.0	88.7	*	***	**
Gentiobiose			27.3	20.4		*	
Glucaric acid	3.7	3.0	16.1	10.4	n.s.	*	n.s.
Glucose	41843.5	28552.0	16547.4	6251.4	*	***	***
Glycerol‐3‐p	82.4	30.8	674.0	362.7	*	***	***
Isomaltose	75.1	0.0			**		
Itaconic acid	142.0	32.2			*		
Maltose	1119.8	678.6	1081.4	846.2	*	*	***
Melibiose	0.0	5.4			***		
Methylmaleic acid			16.7	7.1		*	
Pyruvic acid	42.4	38.6	59.8	44.0	n.s.	**	n.s.
Raffinose	477.5	322.5	218.9	152.7	*	n.s.	**
Sedoheptulose	853.9	782.4	452.2	312.6	n.s.	**	n.s.
Sorbitol	17.0	39.5			*		
Turanose			13688.2	7035.2		**	
Xylitol	18.6	11.5	13.7	20.4	n.s.	*	n.s.
Xylose	165.8	81.1	64.6	49.3	*	n.s.	*
Fatty acids							
13‐eicosenoic acid			5.7	7.6		*	
7‐hexadecenoic acid			132.2	27.0		*	
9,12‐octadecadienoic acid			6032.7	7434.6		*	
Docosanoic acid	50.5	241.3	176.5	1224.1	**	***	***
Hexacosanoic acid	13.2	28.9	0.0	20.9	*	***	***
Hexadecanol	0.0	12.2			***		
Octacosanol	9.5	1.9			**		
Tetracosanoic acid	1206.3	5001.7	8103.8	16232.9	*	*	**
Tetracosanol	2.2	0.0	2.5	4.5	***	**	n.s.
Tricosanoic acid	0.0	12.6	133.6	150.7	**	n.s.	n.s.
Tritriacontanol			47.1	37.6		*	
Amino acids							
Alanine			2.7	6.8		**	
Aspartic acid			928.9	2095.8		*	
Glycine			13.2	57.0		*	
N‐acetylglutamic acid			158.0	1221.7		*	
Serine			216.4	818.2		*	
Threonine	0.0	3.8	311.1	383.3	***	*	*
Valine	0.0	10.3	104.9	92.8	**	n.s.	n.s.
Others							
1,2,3‐hydroxybutane	4.1	0.0			*		
2,3‐dihydroxybutanedioic acid	11.8	27.0	5.8	9.6	*	*	**
2,4,6‐tri‐tert.‐butylbenzenethiol	42.5	44.3	157.3	185.3	n.s.	*	n.s.
2‐aminoethylphosphate			0.0	44.5		*	
2‐desoxy‐pentos‐3‐ulose	33.1	15.3			***		
2‐hydroxybutanoic acid			11.3	0.0		*	
2‐o‐glycerol‐galactopyranoside	2362.0	398.0	181.6	178.0	***	n.s.	*
2‐oxo‐3‐hydroxypropanoic acid			17.3	75.7		**	
3‐methylthiopropylamine			0.0	5.4		*	
5‐o‐coumaroyl‐d‐quinic acid			38.5	15.6		*	
Aconitic acid	202.7	103.5	12.7	37.5	*	*	n.s.
Tocopherol	7.8	4.0			*		
Chlorogenic acid			710.6	332.2		*	
Erythronic acid‐1,4‐lactone			24.2	38.0		*	
Ferulic acid	11.3	30.4	41.8	61.6	n.s.	**	**
Hydroxylamine			39.2	27.1		**	
Ketomalonic acid			0.0	3.1		*	
Malonic acid	59.7	89.5	0.0	2.6	n.s.	**	n.s.
P‐hydroxyacetophenone	0.0	2.8			***		
Protocatechuic acid			5.9	2.1		*	
Putrescine			0.0	22.4		**	
Stigmasterol	40.6	52.0	29.5	9.1	n.s.	**	n.s.
Vanillin	0.0	1.8			***		

Data are means (*n* = 4). Empty indicates absence or undetected metabolites. Genotypes are significant different at *p*‐Value < .05 (*), 0.01 (**), and 0.001 (***) based on two‐tailed *t* test in Experiment 1 (E1), Experiment 2 (E2), or nested (*N*) analysis of two experiments combined together. Carbohydrates indicate sugar‐related compounds and derivatives in citric acid cycle. Fatty acids indicate fatty acids and their derivatives. Others indicate all other compounds not belong to carbohydrates or fatty acids, or amino acids.

Based on maltose and glucose relative concentrations, the estimated starch concentration in SH was 1.7 times that of YK over the two experiments. Furthermore, the products of starch degradation were more abundant in SH than YK in both profiling experiments (Table 3). Maltose and glucose, primary products of starch degradation, were elevated in SH along with fructose, glycerol‐3‐phosphate, raffinose, and an unresolved disaccharide. This suggested more active 6‐carbon metabolite catabolism via glycolysis in SH compared to YK during the night (Table 3; Table S3). SH accumulated higher concentrations of the disaccharides isomaltose and gentiobiose, whereas YK accumulated a very small amount of melibiose in one of the two screens. Tricarboxylic acid (TCA) cycle intermediates, on the other hand, were not consistently different between the accessions. Citric acid and fumaric acid were accumulated at higher levels in YK, and alpha‐ketoglutaric acid was greater in SH (Table 3; Table S3). Ascorbic acid was higher in SH, but the ascorbic acid degradation product tartaric acid (2,3 dihydroxybutanedioic acid) was higher in YK (Table 3 and Table S3).

All amino acids that differed between SH and YK were higher in YK (Table 3; Table S3). These included alanine, glycine, serine, threonine, and valine. Of these, only threonine was differentially accumulated in both profiling experiments. The amino acids alanine, glycine, and serine were only detected in one of the two experiments. Valine was detected in both experiments, but the difference between SH and YK was only statistically significant in the first experiment. Concentrations of the other detected amino acids (isoleucine, leucine, and proline) were not different in the two accessions.

Pathway analyses were conducted on both transcriptome (Table 2) and metabolome data (Table 3) to identify differences between SH and YK during the dark period and any correlation between transcriptome and metabolome analyses of the two accessions. The metabolite pathway analysis identified two functional categories: major carbon degradation (downregulated in YK) and amino acid synthesis (upregulated in YK). Glucose, maltose, and isomaltose are within the major carbon degradation pathway, while aspartic acid, alanine, N‐acetylglutamic acid, threonine, serine, and glycine are within the amino acid synthesis pathway. However, of the 19 DEGs annotated in the reference genome, one unknown functional category including nine DEGs (Thhalv10015083 m.g, Thhalv10002969 m.g, Thhalv10023491 m.g, Thhalv10021522 m.g, Thhalv10018393 m.g, Thhalv10009345 m.g, Thhalv10015718 m.g, Thhalv10022932 m.g, and Thhalv10022943 m.g) and one category related to cell organization of two DEGs (Thhalv10029390 m.g and Thhalv10029346 m.g) were identified. Hence, our analyses did not show any correlation between the *E. salsugineum* transcriptome and metabolome.

### 3.4 Metabolomic phenotypes in the F_1_ hybrid exhibit transgressive heterosis

To better understand the genetic basis for observed differences in metabolite concentrations between SH and YK, we measured metabolites in F_1_ hybrids. Concentrations of all measured metabolites in both experiments are presented in Table S3. Of the 144 metabolites measured in the experiment comparing the two parents and hybrids, 56% of all metabolites were lower in hybrids than the predicted mid‐parent value. This included 65% of the fatty acids and 49% of carbohydrates detected (Table S3). We performed a two‐way contingency test to determine if an observed difference in the accumulation of a metabolite was predictive of heterosis for that metabolite (Table S7). We found that metabolites with accumulation differences between the parents were neither more nor less likely to exhibit accumulation differences between the F_1_ and the mid‐parental values (Table S7).

**Figure 1:**
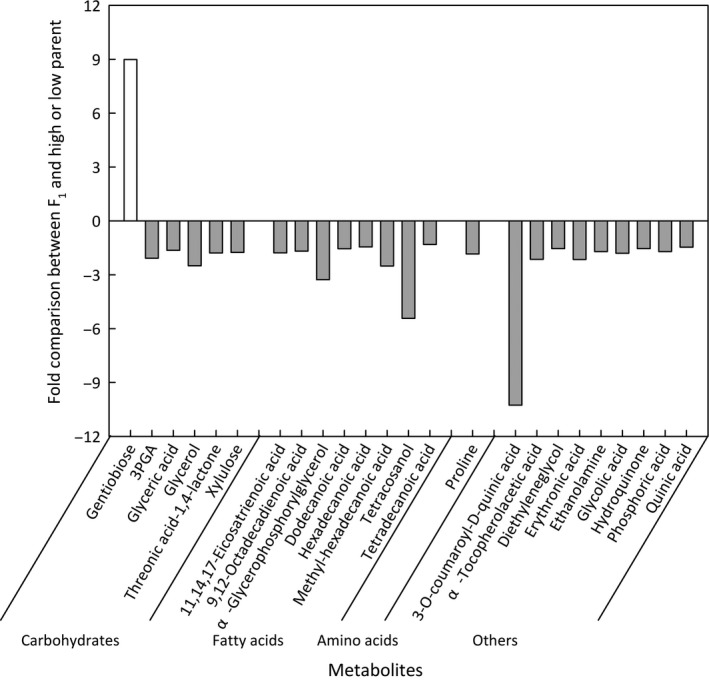
Heterosis for metabolite concentrations in F_1_ hybrids. Metabolites in F_1_ hybrids that were higher than the high parent or lower than the low parent at *p*‐value ≤ .05 based on two‐tailed *t* test are shown. The ratio is calculated as F_1_/high parent when F_1_ had a higher concentration than the high parent and low parent/F_1_ when F_1_ had a lower concentration than the low parent

Hybridization can result in transgressive heterosis in which phenotypic values for the hybrids fall outside of the range of parental values. Of the 144 metabolites measured, transgressive heterosis was observed for 28. Of these, four metabolites were not observed in one of the two parents and 24 were detected in both parents and the hybrids (Figure 1). Of the 24 detected in all three genotypes, the transgressive heterosis was more likely to be negative (Figure 1; *p*‐value ≤ .0001 based on a Binomial exact test) and more frequently affected metabolites that did not differ in concentration between the parents (Table S7; *p*‐value ≤ 0.05 based on χ^2^ test). Thus, heterosis for the metabolome was manifested by a decrease in metabolite pool sizes in hybrids and was not preferentially associated with metabolites that contributed to the variation between the two parents. This is consistent with our observation of decreased availability of primary metabolites in the faster growing YK (Table 3; Table S3). We propose that a metabolic consequence of enhanced growth is a reduction in pool sizes of primary metabolites and greater resource utilization for anabolic metabolism.

## 4 DISCUSSION

In this study, we profiled both transcript and metabolite accumulation to identify genetic and biochemical variation during the dark period in two *E. salsugineum* accessions: SH and YK. We annotated novel genes present in the reference genome and identified DEGs between SH and YK in the middle of the dark period. We found that YK accumulates more fatty acids than SH, while SH accumulates sugars at higher concentrations. Although the transcriptomic and metabolic profiling results do not offer links to each other, they do offer insight into genetic and physiological differences between these accessions. Furthermore, we identified additional SNPs and provide validation of previously described SNPs, varying between these accessions that can be utilized for future research.

### 4.1 Validity of identified SNPs

Based on the predicted transcriptome size (Yang et al., 2013), the SNP density from our analysis is 1 SNP per 10 kb of transcribed sequence; 14,887 SNPs identified from our transcriptome sequencing data were also present in Champigny et al. (2013). However, our SNP cluster filter, which removes neighboring SNPs to account for misaligned reads at insertion–deletion polymorphisms, removed 3,604 overlapping SNPs. The removal of this set of SNPs, likely enriched for false positives, contributed to the lower SNP number detected in our study. We also acknowledge that this contributed to our false negative rate. Of the 388 SNPs that matched between our analyses of pyrosequencing data and the Sanger data, 99 were removed by our procedure. We provide a compact file containing the subset of “high‐quality” (see Materials and Methods) SNPs in Table S6. These have a SNP density of one SNP per 25 kb of transcript. This set of validated SNPs can be used for QTL mapping and fine‐mapping studies (Matsuda et al., 2015; Trick et al., 2012; Wu et al., 2016).

### 4.2 Identification of genes expressed in the dark period in *Eutrema salsugineum*

We provided expression data supporting 63% and 67% of the predicted genes in the two published reference genomes (Wu et al., 2012; Yang et al., 2013). This is likely an underestimate of the expressed genes in this species because we sampled only rosette tissue and only at night (Schaffer et al., 2001). We identified 66% of the genes identified in another transcriptome analysis of *E. salsugineum* (Champigny et al., 2013). Despite our study relying on lower coverage (4× vs. 8×), and quantifying expression of genes only expressed in leaves at night, we annotated 65 genes not predicted in the reference genome or identified in the previous transcriptome characterization (Tables S1 and S5). Novel genes identified in this transcriptome study but not that of Champigny et al. (2013) are likely either only expressed at night or otherwise not expressed under the conditions of the previous study, which included a 21‐hr photoperiod and low dark period temperatures.

More than 97% of the genes identified in our study are homologous to genes in *A. thaliana* (Table S1), consistent with the relatively close phylogenetic association of the two species (Al‐shehbaz, O'Kane, & Price, 1999). Of the 65 newly annotated genes in this study, 47 of them do not have homologs in *A. thaliana*. One of these novel genes has been annotated in *A. lyrata* and two have been annotated in *S. parvula* (Table S1). Therefore, we may have identified genes that are unique to this extremophile species (Wu et al., 2012).

More than 80% of the expressed genes identified in this study were detected in both SH and YK (Table S5). This indicates a conserved transcriptome between these two accessions and is consistent with previous *E. salsugineum* transcriptome comparisons (Champigny et al., 2013; Lee et al., 2013). PAVs, as scored by read count in the transcript profiling experiment, accounted for 19 and 12% of all genes in this experiment and Champigny et al. (2013), respectively. Only 13% of these were consistently detected as PAV over the two datasets. These genes are likely “true” PAV genes (Table S1). Other genes detected in only one of the replicates were likely due to low mRNA abundance. PAV structural variation has been observed in *A. thaliana* (Bush et al., 2014), maize (Springer et al., 2009; Swanson‐Wagner et al., 2010), and soybean (Haun et al., 2011). PAV genes are not typically essential (Bush et al., 2014) and may have minor effects on plant fitness (Swanson‐Wagner et al., 2010). However, genes present in only one accession could contribute to the adaptation to specific selective constraints (Bush et al., 2014) and variation in quantitative traits (Swanson‐Wagner et al., 2010). The observed phenotypic variation in growth rate and metabolism could be due to PAV, although no identified PAVs have been linked to trait differences in *Eutrema*. Further study utilizing molecular genetics to address the causes and consequences of natural variation in this species is needed to link these data types.

### 4.3 DEGs and constitutive response to abiotic and biotic stresses

The low number of candidate DEGs between the two accessions of *E. salsugineum* used in this study was similar to other studies; 55% of these had no reads in one of the two accessions, which could be due to a lack of sufficient depth to detect very low expression. Champigny et al. (2013) identified 381 DEGs that were present in our transcriptome but not identified as DEGs in our data. RNA‐seq experiments are often underpowered, and the lack of overlap with the previous study may be primarily an issue of low read depth and replicate numbers. Consistent with this expectation, 58% of the 381 DEGs identified previously have low expression (<4 reads; Table S1). In addition, 41% of the DEGs from Champigny et al. (2013) exhibit lower expression during the dark period in *A. thaliana* (Mockler et al., 2007), which may explain some of the lack of overlap in DEGs in the two studies. We identified 17 DEGs that were not identified by Champigny et al. (2013), possibly because they are only differentially expressed during the dark period or under our experimental conditions.

It appears that the relative abundance of most transcripts is similar in SH and YK, as only 0.2 and 1.9% of all genes were DEGs in our study and Champigny et al. (2013), respectively. Fourteen of the 31 DEGs identified in our study were also identified by Champigny et al. (2013), and the expression patterns were the same in both studies. This suggests that differential expression for these genes between SH and YK is consistent over light and dark periods and the two growth conditions.

Without exposure to abiotic or biotic stresses, YK expressed several stress‐responsive genes (Table 2), as also noted by Champigny et al. (2013). Two plant defensin genes within the same family were highly expressed in YK (Table 2). In *A. thaliana,* the plant defensin type 1 family (*PDF1*) is comprised of seven genes (Shahzad et al., 2013) with highly conserved sequences and identical mature peptides (Thomma, Cammue, & Thevissen, 2002). *AtPDF1* genes are induced by pathogens, nonhost pathogens, methyl jasmonate (MeJA), and ethylene (ET; De Coninck et al., 2010; Manners et al., 1998; Penninckx et al., 1996; Zimmerli, Stein, Lipka, Schulze‐Lefert, & Somerville, 2004). Also, expression of *AtPDF1* genes in yeast results in zinc tolerance (Shahzad et al., 2013). Eight gene models in *E. salsugineum* are homologous to the *A. thaliana* plant defensin family. Of these, one is a likely ortholog of *AtPDF1.4*, whereas five are annotated as most closely related to *AtPDF1.2A*, and two are annotated as *AtPDF1.2C*. Higher expression of *PDF1.2* and *PDF1.4* was observed in the YK accession; however, paralogous *PDF1* genes are expressed in SH (Table S1).

The transcript abundance of a gene encoding *E. salsugineum* peptide methionine sulfoxide reductase 3 (*PMSR3*) was higher in YK than SH (Table 2). There are five orthologous *PMSR* genes in *A. thaliana*,* PMSR1* to *5* (Rouhier, Vieira Dos Santos, Tarrago, & Rey, 2006), that are also found in *E. salsugineum*. The expression of *PMSR3* is induced by arsenate (Paulose, Kandasamy, & Dhankher, 2010). No function in tolerance or resistance has been established for this paralog in *A. thaliana*. However, knockout of either *PMSR2* (Bechtold, Murphy, & Mullineaux, 2004) or *PMSR4* (Romero, Berlett, Jensen, Pell, & Tien, 2004) results in decreased oxidative stress tolerance and overexpression of either gene increases stress tolerance in *A. thaliana*. Expression of *PMSR4* (but not *PMSR1*,* PMSR2*, or *PMSR3*) was induced in response to UV and AgNO_3_ in *E. salsuginea* SH (Mucha, Walther, Muller, Hincha, & Glaswischnig, 2015). It is plausible that the overexpression of *PMSR3* by YK could provide greater oxidative stress tolerance in this accession.

### 4.4 *Eutrema salsugineum* accessions SH and YK differ in carbon metabolism

In two experiments, 125 and 144 metabolites were detected. Although the total number and specific metabolites varied somewhat across the two experiments, differences were identified between SH and YK for the 85 metabolites detected in both experiments (Table 3; Table S3). Differences across the experiments may be due to slight differences in the growth chamber environments even with identical settings, as metabolite concentrations are strongly affected by environmental conditions and environment by genotype interactions (Soltis & Kliebenstein, 2015). Also, different soilless media mixes were used in the two experiments: A more bark‐based media was used in the transcriptome and first metabolite experiments, whereas the soilless media used in the second experiment did not contain bark. However, there were consistent growth differences between the two accessions, and we focused our interpretation of the data primarily on those metabolites that were consistent across the two experiments.

The derivatization method we utilized has been widely used to detect sugars (Gullberg et al., 2004), but is less accurate for identifying and quantifying amino acids (Kaspar, Dettmer, Gronwald, & Oefner, 2009). As a result, despite the fact that *E. salsugineum* accumulates higher concentrations of some amino acids than Arabidopsis (Eshel et al., 2017), many amino acids were not detected in our analyses.

Higher concentrations of fatty acids and fatty acid derivatives were measured in YK, including several previously identified as structural components of membrane lipids, cuticle components, and wall‐resident suberin (Table 3; Table S3). Fatty acids contain more energy than carbohydrates when used as storage compounds and can act as an efficient storage form of reduced carbon (Taiz & Zeiger, 2010). High production of fatty acids is typical of rapidly growing tissues (Ohlrogge & Jaworski, 1997; Qin et al., 2007). Very‐long‐chain fatty acids (VLCFAs; C20:0 to C30:0) play an important role in cell elongation and expansion (Qin et al., 2007). Several VLCFAs, including docosanoic, hexacosanoic, pentacosanoic, tetracosanoic, and tricosanoic acids, were more abundant in YK than SH (Table 3), consistent with our measurements of higher growth rates of YK as compared to SH in our growth conditions (manuscript in preparation). The VLCFA tetracosanoic acid, which plays an important role in root cell growth and expansion (Qin et al., 2007), was accumulated at a higher concentration in YK (Table 3; Table S3). In addition to carbon storage, lipids are important components of membranes and the leaf cuticle (Lynch & Dunn, 2004; Tresch, Heilmann, Christiansen, Looser, & Grossmann, 2012; Zäuner, Ternes, & Warnecke, 2010). Our results are in agreement with Xu et al. (2014), who measured a greater accumulation of C22 and C24 fatty acids in the epicuticular wax of YK over SH.

Overall, SH tissues had higher concentrations of sugars than YK. These measurements of nighttime sugar concentrations were similar to previous results obtained for fructose, glucose, and raffinose in leaves of *E. salsugineum* harvested during the day (Eshel et al., 2017; Lee et al., 2012). The products of starch breakdown, including maltose and glucose, were more abundant in SH (Table 3; Table S3), suggesting a higher rate of starch metabolism in the lower‐biomass SH accession. This was consistent with the strong negative correlation between starch content and biomass observed in *A. thaliana* accessions (Sulpice et al., 2009).

A study of the correlation between specific metabolites and biomass accumulation in *A. thaliana* revealed twenty‐three metabolites that were correlated with biomass (Meyer et al., 2007). We detected fourteen of these twenty‐three metabolites (Table 3; Table S3). Of these, five differed between SH and YK. The concentrations of ascorbic acid, glycerol‐3‐phosphate, and raffinose were negatively correlated, and putrescine was positively correlated with biomass in *Arabidopsis* (Meyer et al., 2007). The levels of these metabolites also corresponded to the differences in biomass between SH and YK, indicating that the relationships between metabolites and biomass found in *A. thaliana* were consistent in *E. salsugineum* (Table 3; Table S3). This suggests that, although stress tolerance is vastly different between these two species (Amtmann, 2009; Griffith et al., 2007; Lee et al., 2012), the metabolic markers for biomass accumulation may be similar.

### 4.5 Increased utilization rate as a hypothesis for metabolome heterosis

More than 58% of the metabolites in F_1_ plants were different from the predicted mid‐parent concentration (Table S3), indicating a nonadditive effect of hybridity on the majority of the metabolome. The lower concentration of fructose and glucose in F_1_ hybrids suggests high rates of starch and sugar depletion to support rapid growth (Lisec et al., 2011). Transgressive heterosis was more commonly observed for metabolites that were not different between the two parents (Table S7). This suggests that allelic variation affecting differential metabolite accumulation in the parents is not responsible for the observed heterosis in the F_1_ metabolome. Although it is surprising that differences between the parents were not predictive of a metabolite association with heterosis, it may be that the metabolomic consequences of heterosis derived from secondary effects of an increased growth rate in F_1_ hybrids, rather than a causative relationship between growth rate and specific metabolites or metabolite diversity. Differences in biomass polymers and metabolites involved in anabolic growth exhibited reduced pool sizes in the more rapidly growing YK as compared to SH (Table 3; Table S3), as well as in the very rapidly growing F_1_ plants as compared to the parents (Table S3). Consistent with the hypothesis that utilization rate drives the heterotic effects on metabolite pool sizes, the transgressive effect overwhelmingly resulted in lower concentrations of metabolites in the hybrids (Figure 1; Tables S3 and S7). This hypothesis regarding the cause of metabolic heterosis may be a general phenomenon in plants. Indeed, the same associations have been observed in maize, in which largely negative overdominance for metabolites was found in the heterotic B73 × Mo17 hybrids (Lisec et al., 2011).

## 5 CONCLUSIONS

Our study contributes to the annotation of the *E. salsugineum* genome and provides evidence of transcriptional and metabolic differences between the SH and YK accessions. Very few differences in gene expression were detected in the middle of the dark period between these two accessions, but YK has constitutively higher expression of several plant systematic defense genes. The high‐quality SNPs identified in this study can be used with previously identified SNPs to map traits that differ in these accessions, such as tolerance to various stresses. There is evidence for contrasting carbon metabolism in these two accessions, which correlates with observed growth differences. Furthermore, metabolite profiling of the accessions and F_1_ hybrids supports the notion that the concentrations of key metabolites are correlated with growth rate, including the increased growth rate caused by heterosis.

Our hypothesis was that combined transcriptome and metabolome profiling of two contrasting *E. salsugineum* accessions might elucidate the pathway(s) related to the phenotypic differences between these contrasting accessions. The difference in carbon metabolism identified via metabolome profiling provides insights for growth differences between SH and YK. However, none of the 19 DEGs that have been annotated in the reference genome are related to the observed metabolic differences. There are two plausible explanations: (i) the additional 11 DEGs that are currently unannotated in the reference genome could provide additional evidence for the link between metabolome and transcriptome, or (ii) by increasing the number of replicates in transcriptome study, more DEGs will be identified to support further pathway identification.

## COMPETING INTERESTS

The authors declare that they have no competing interests.

## AVAILABILITY OF SUPPORTING DATA

All the raw data supporting the results of this article have been deposited at Edgar, Domrachev, & Lash (2002) and are accessible through GEO Series accession GSE GSE67745 at (http://www.ncbi.nlm.nih.gov/geo/query/acc.cgi?acc=GSE67745).

## AUTHORS' CONTRIBUTIONS

JY and MJG performed the experiments; JY, BPD, and MVM designed the experiments; JY and BPD analyzed the data; JY, BPD, and MVM wrote the manuscript.
