# Supplementary material for: Dark period transcriptomic and metabolic profiling of two diverse Eutrema salsugineum accessions
Source: Plant Direct. 2018 Feb 22;2(2):e00032. doi: 10.1002/pld3.32 (PMC6508522; doi:10.1002/pld3.32)
Supplement: Supplementary file 2 [file PLD3-2-e00032-s002.docx]

| **Supplementary Table 2. Primer sequences used for qRT-PCR. Gene name (Name) and primer sequence (Sequence) for all genes used to test transcriptome expression differences by qRT-PCR.** | |
| --- | --- |
|  |  |
|  |  |
| **Name** | **Sequence** |
| Thhalv10015718m.g-QRTF | ACCAGCTCCATTTGGATTGAAA |
| Thhalv10015718m.g-QRTR | GGAGTCCCCTTTGTCTTCGAT |
| Thhalv10022994m.g-QRTF | GCCCGGTGGTGGTGATAAG |
| Thhalv10022994m.g-QRTR | GCCACAAACCCATTCCTCAA |
| Thhalv10029390m.g-QRTF | TTACGCCAAATAACACCCAACA |
| Thhalv10029390m.g-QRTR | TCCCGAAATCACCTGACACA |
| Thhalv10021522m.g-QRTF | GCCTCACCGCACCGTTAA |
| Thhalv10021522m.g-QRTR | GGACTAAACTGAAGAGGCCAGAGA |
| Thhalv10021382m.g_SH-QRTF | ATCTCCTGGAAATGCGACTGA |
| Thhalv10021382m.g_SH-QRTR | TGCTAGGTTGGGCGAGATG |
| Thhalv10021382m.g_YK-QRTF | CCCTGGAAAGACGACTGACAAG |
| Thhalv10021382m.g_YK-QRTR | GGTGGGCGAGATTGTGATTG |
| Thhalv10014718m.g-QRTF | GGGTCGGTGCAGCCTTT |
| Thhalv10014718m.g-QRTR | GAGGAATATCATCAGCAGTACCTTTCA |
| Thhalv10002969m.g-QRTF | CGTACGTTTCTTTCGCTTATTGG |
| Thhalv10002969m.g-QRTR | TGGCCACAGCATGAGATCAG |
| Thhalv10000662m.g-QRTF | TGGCTTCCTTCATCGCTCTT |
| Thhalv10000662m.g-QRTR | TCGCACAACTTCTGAGCTTTCA |
| Thhalv10022932m.g-QRTF | CTTGGCGCCCGAGTACAG |
| Thhalv10022932m.g-QRTR | CACGCAGGCGATTTTGG |
| Thhalv10014933m.g-QRTF | CGCTGCGAGAGACAACAAGA |
| Thhalv10014933m.g-QRTR | GCGATTGTGACTCCACTGAGAA |
| Thhalv10019398m.g-QRTF | GCAGGGAAGACATAGTTGCAAGAT |
| Thhalv10019398m.g-QRTR | AAGTGGGACATGGTCTGGTGTT |
| Thhalv10009345m.g-QRTF | CACTTCTTCTCCAAATTCTCGATCT |
| Thhalv10009345m.g-QRTR | GCATCGGGTCGGAGAAATC |
| Thhalv10014264m.g-QRTF | GCACCGGAAGAAGTTGGAGAT |
| Thhalv10014264m.g-QRTR | CTGCCCAAAGCTCTTGATCAT |
| Thhalv10000285m.g-QRTF | GGGCTGGGTGTGATTTTCC |
| Thhalv10000285m.g-QRTR | AGGGCGAGCGTGGTACAGT |
| Thhalv10022943m.g-QRTF | TTTGATCGGATTTTCTTCGGTATAT |
| Thhalv10022943m.g-QRTR | AATTCTGATGGAAACAGATTAAACGA |
| Thhalv10023491m.g-QRTF | GAATTTGAGTATGACGAGGATGCA |
| Thhalv10023491m.g-QRTR | TTGTCCACAGAAGAATGTCCATTT |
| Thhalv10015083m.g-QRTF | ACCGCCACTTGACCAGGAT |
| Thhalv10015083m.g-QRTR | CTTCCATGCTTGTCGTTCTTCTT |
| Thhalv10018393m.g-QRTF | TTCTTCACCCTTCTCGTTCTCAT |
| Thhalv10018393m.g-QRTR | GGGCTTTTGCTGGTCATACTG |
| Thhalv10029246m.g-QRTF | TGAGCCACATGATGCAAAGG |
| Thhalv10029246m.g-QRTR | TCGAATGGTGAGAGGGTTTGT |
| Thhalv10011087m.g-QRTF | CAAATCATCGGTCCGGTACTG |
| Thhalv10011087m.g-QRTR | CCCTTAACCACCAGAGCATTG |
| XLOC_005768-QRTF | TGTGTGTTAATCCTCTGCAGATCTATC |
| XLOC_005768-QRTR | GCCATGGAAGGCATCTTCA |
| XLOC_017575-QRTF | TCAACGAGATTCCGAGGTGTAA |
| XLOC_017575-QRTR | CTGCGAATCCGATAAGCTAGGA |
| XLOC_003055-QRTF | GGAGTTTACTACGGCGATGCA |
| XLOC_003055-QRTR | CATTACAATCTGGATCAACACAAAGA |
| XLOC_024727-QRTF | GCGTTTGCGTTTGATTGCT |
| XLOC_024727-QRTR | ACCAAGCTCAACACGACAAGAA |
| XLOC_003052-QRTF | CACAAAAAAACGACAACAGAAACCT |
| XLOC_003052-QRTR | GGCTTGCTGGTTTAGATTCTTAGG |
| XLOC_008740-QRTF | CAGTCAAGAGAGGATAAAACCCAAA |
| XLOC_008740-QRTR | CTCTTCCGCATATGAACTCCTCTTA |
| XLOC_020731-QRTF | TGTTGGATTTGTTTTCTGGAATCA |
| XLOC_020731-QRTR | CCGCTTATCCCCACCTCAA |
| XLOC_017573-QRTF | ACCGGATCTGAAGGCATCAC |
| XLOC_017573-QRTR | CGCTTATATCGGTTCCGACTTG |
| XLOC_015175-QRTF | CACCGGAATTTCGATCTGTTACT |
| XLOC_015175-QRTR | GTGATACACGTGTGGGTCAAAGA |
| XLOC_024729-QRTF | GAAATCAAATAGAGAAAACAAGTACGAAGT |
| XLOC_024729-QRTR | ACTGGGACTCGTCTTTATCACATTC |
|  |  |
