# Supplementary material for: Dark period transcriptomic and metabolic profiling of two diverse Eutrema salsugineum accessions
Source: Plant Direct. 2018 Feb 22;2(2):e00032. doi: 10.1002/pld3.32 (PMC6508522; doi:10.1002/pld3.32)
Supplement: Supplementary file 4 [file PLD3-2-e00032-s004.docx]

| **Supplemental Table 4** **Number of reads, percentage of reads uniquely aligned to reference genome (%), mean read length (bp) of *E. salsugineum* Shandong (SH) and Yukon (YK) accessions** | | | |
| --- | --- | --- | --- |
|  | | | |
|  | | | |
| **Accession** | **Total reads** | **Uniquely aligned (%)** | **Mean read length**  **(bp)** |
|  | |  |  |
|  | |  |  |
| SH | 541,494 | 95.6 | 365.8 |
| YK | 491,926 | 94.4 | 361.8 |
| Total | 1,033,420 | 95.0 | 363.8 |
|  | | | |
