# Supplementary material for: Dark period transcriptomic and metabolic profiling of two diverse Eutrema salsugineum accessions
Source: Plant Direct. 2018 Feb 22;2(2):e00032. doi: 10.1002/pld3.32 (PMC6508522; doi:10.1002/pld3.32)
Supplement: Supplementary file 5 [file PLD3-2-e00032-s005.docx]

| **Supplemental Table 5 Summary of transcriptome assembly of *E. salsugineum* Shandong (SH) and Yukon (YK) accessions** | |  |
| --- | --- | --- |
|  | |  |
|  | |  |
| Number of assembled genes | 17,888 |  |
| Shared | 14,577 |  |
| SH only | 1,643 |  |
| YK only | 1,668 |  |
|  |  |  |
| Newly annotated genes | 157 |  |
| Shared | 122 |  |
| SH only | 26 |  |
| YK only | 9 |  |
| Annotated by best blast hit from *A. thaliana*, *A. lyrata* and/or *E. parvulum* | 54 |  |
| Newly annotated genes unique from genes annotated by Champigny et al., 2013 | 65 |  |
| Newly annotated genes present in genes annotated by Champigny et al., 2013 | 92 |  |
|  | | |
