# Supplementary material for: Dark period transcriptomic and metabolic profiling of two diverse Eutrema salsugineum accessions
Source: Plant Direct. 2018 Feb 22;2(2):e00032. doi: 10.1002/pld3.32 (PMC6508522; doi:10.1002/pld3.32)
Supplement: Supplementary file 7 [file PLD3-2-e00032-s007.docx]

| **Supplementary Table 7 Heterosis effect of metabolites in SH x YK F_1_ hybrids of *E. salsugineum*** | | | | |
| --- | --- | --- | --- | --- |
|  | | | | |
|  | | | | |
|  | F_1_ different from mid-parent | | F_1_ not different from mid-parent | |
|  |  | |  | |
|  |  | |  | |
| SH different from YK | | 31 | 23 | |
| SH not different from YK | | 53 | 37 | |
| Chi-square | 0.03 | | | |
| *P-*value | n.s. (0.8614) | | | |
|  | F_1_ > high parent or < low parent | | | F1 between the two parents |
|  |  | |  | |
|  |  | |  | |
| SH different from YK | | 5 | 49 | |
| SH not different from YK | | 23 | 67 | |
| Chi-square | 5.72 | | | |
| *P-*value | * (0.01675) | | | |
|  |  | |  | |
|  |  | |  | |
|  | Observed | |  | |
|  |  | |  | |
|  |  | |  | |
| F_1_ > high parent | 1 | |  | |
| F_1_ < low parent | 23 | |  | |
| *P-*value | *** (2.98 x10^-6^) | |  | |
|  |  | |  | |
