# Supplementary material for: Dark period transcriptomic and metabolic profiling of two diverse Eutrema salsugineum accessions
Source: Plant Direct. 2018 Feb 22;2(2):e00032. doi: 10.1002/pld3.32 (PMC6508522; doi:10.1002/pld3.32)
Supplement: Supplementary file 8 [file PLD3-2-e00032-s008.pdf]

Dear Ana,

We have discussed your comments and our joint responses are below. We hope this will resolve any remaining issues. Please let us know if you need anything else from us.

Sincerely,
Mike and Brian

Dear Michael,

The reviewer did not receive your responses but I contacted him and he said the changes were not accommodated which I agree and that's why I sent the paper for your revision. I should have made clearer that the letter issue was not the main problem and I apologize for that. But I am not planning to send the article for another round of revision and that´s why I move it forward. In fact, the changes suggested were very simple to be performed but as I noted yesterday they were not in fact carried out.
So I will go through the points raised by the referee:

1- Too many references cited; perhaps this could be reduced to a smaller amount.
As I checked the authors did not reduce a single reference. As an example in the introduction:" *Eutrema salsugineum* (formerly *Thellungiella halophila*) is a model species for the study of plant stress tolerance (Amtmann, 2009; Bressan et al., 2001; Griffith et al., 2007; Oh et al., 2010; Orsini et al., 2010; Pilarska et al., 2016; Taji et al., 2004; Volkov et al., 2003; Wong et al., 2005)."
I don´t think the authors need this amount of references for this statement.

We have removed 14 references from the manuscript that we deemed as not required for context or supporting information for conclusions drawn. We hope this will be satisfactory.

As we noted in our response to reviewer #2's comment, we acknowledge that there are a large number of references in the manuscript. We are surprised that a less complete citation of the literature is preferred by the reviewer and by the journal. Regardless, the results of the transcriptomic and metabolomics analyses require some discussion of a large number of genes, gene products, and metabolic pathways, and as a result many references to previous work. In the example above, these cited works established this species as an important model system for the investigation of plant responses to the environment. Many readers will not be familiar with this species, and previous work. These papers represent foundational studies on this species, including the high tolerance to multiple stresses.

2- Not sure Tables 1 and 2 should be presented within the main text (better suited as Supplementary material).

This is obviously subjective but I also think that these days these kind of information should go to Supplemental material.

We agree that this is a subjective choice. We do not object to moving the tables to the supplemental and have done so. All callouts to these tables in the text now refer to the supplemental materials.

3- I would have liked to see a GO enrichment Table from the RNAseq analysis.

This was not done either and the authors justify that given the small number of DEGs, GO analysis was not appropriate. I do not agree that is not appropriate, it can be done, but obviously the information obtained can be very limited. I think the reviewer raised this point to have further insights into the functional category of the differentially expressed genes (e.g Plant defensin/ stress response) and eventually the manuscript could be less descriptive. This again in my opinion is not a major issue but I understand reviewer´s suggestion. Anyhow, it will not be required for publication.

As stated in our initial response to the reviews, there were a very small number of DEGs between the two accessions. For this reason, a GO analysis is not informative. We thank you for not making this a required modification.

4- Also, the way RNAseq data are presented is rather unusual. Nowadays it is more reliable to use log2FC values.
This suggestion was accommodated by the authors.

5- Metabolite data are quite different between experiments for certain cases (metabolites). Could you please provide a plausible explanation for this discrepancy?

The authors partially addressed this issue by adding: "*In two experiments, 125 and 144 metabolites were detected. Although the total number and specific metabolites varied somewhat across the two experiments, differences were identified between SH and YK for the 85 metabolites detected in both experiments (Table 5 and Supplementary Table 3). Differences across the experiments may be due to slight differences in the growth chamber environments even with identical settings, as metabolite concentrations are strongly affected by environmental conditions and environment by genotype interactions (Soltis and Kliebenstein, 2015). Also, different soilless media mixes were used in the two experiments: a more bark-based media was used in the transcriptome and first metabolite experiments, whereas the soilless media used in the second experiment did not contain bark. However, there were consistent growth differences between the two accessions and we focused our interpretation of the data primarily on those metabolites that were consistent across the two experiments.*"
It is unclear why the authors used different media for the two experiments but since there were consistent growth differences between the two accessions this aspect was ignored. It is however, true that the discussion could have a clearer message since the differences in metabolites between the two experiments are really noteworthy. Additionally, the discussion section entitled "*E. salsugineum* accessions SH and YK differ in carbon

metabolism" covers sugars, amino acids, fatty acids and polyamines among others. As the authors acknowledged "Fatty acids contain more energy than carbohydrates when used as storage compounds and can act as an efficient storage form of reduced carbon". So why not to talk about lipid metabolism instead?

We did discuss lipid metabolism and chose to do so at a level that we feel adequately addresses the potential role of differences in lipid metabolism between the two accessions without unwarranted speculation, given the limited amount of data. We hope this is acceptable.

I hope that now I wrote it clearly and I am sorry for the fact that your letter did not reach the reviewer which we will solve soon.

We would appreciate it, since we do not know the reviewer but they know us, if you would please communicate to them that:

1. They evaluated a primary submission and not a revised manuscript despite the Plant Direct system indicating to them that the manuscript was a revision due to a bug.
2. That they were not provided the response to reviews, when we submitted our revision in response to their comments, not due to our negligence or arrogance, but due to a bug in the system.

Bugs like these run the risk of damaging reputations, as we fear may have happened in this case.

Thank you for your time addressing this manuscript,

Mike and Brian
